# Points from Hospital Reports

**Published:** 1924-12

**Authors:** 


### POINTS FROM HOSPITAL REPORTS

### Manchester Royal Infirmary.

The following, quoted almost *verbatim* from the report, refers to the most interesting event in a very busy year. The year 1923 will remain memorable in Manchester by reason of the fact that the Lord Mayor, Councillor William Cundiff, on his own initiative, and by his generous example and courage, raised no less than £74,000 for the benefit of the voluntary hospitals of Manchester. When it is remembered that 1923 was a year of unparalleled trade depression, the magnitude of the achievement will be appreciated. It is gratifying to know that the organisation which achieved this result will continue to exist, and that through the medium of the Lord Mayor's Fund the voluntary hospitals of Manchester will benefit by the activities of a Committee who will endeavour to sustain the enthusiasm which has been created, and to maintain a constant flow of charity from sources which had hitherto been untapped.

### Glasgow Royal Infirmary.

A volume of work not only enormous in itself but greater than ever before, and a financial position at the end of the year eminently satisfactory, are at once testimony to the efficient conduct of the Infirmary and to the public spirit of the people of Glasgow. This sums up the record of the year 1923, and as it carries with it the assurance of perseverance it leaves nothing to chance, and therefore nothing to be desired. An addition to the Nurses' Home, comprising 100 bedrooms and ample lounge and recreation rooms, is in progress. Once the shape of an annual report has been adopted it is apt so to remain, if for no other reason than that change almost inevitably involves expense. Nevertheless we venture to comment on the unsociable, so to speak, size of this report—quarto. Anyone who has made a collection of hospital reports must have formed a very decided opinion of the convenience, to say the least, of uniformity in size. We could wish that every hospital committee would decide to adopt demy-octavo as the size of its annual report, whether it consists of 10 pages or 200.

### Monkwearmouth and Southwick Hospital, Sunderland.

During 1923 there was a slight diminution in the number of in-patients, but as on the whole the cases were of a more serious nature the average daily number in the wards was practically the same as in 1922. There was an increase in the number of out-patients. Unfortunately there was a deficit on the year's working of £1,687, which, relatively to the annual expenditure (£6,538), is a serious figure, about which the Committee is gravely concerned. The statistical tables contain two which are not very common—statements of the employers of in-patients and out-patients. In the case of in-patients the number of days in hospital is given, and in that of out-patients the number of attendances. It is therefore quite easy for the reader, with the aid of the averages of cost stated elsewhere, to calculate the cost to the hospital of the patients sent by any particular firm. Alphabetical arrangement and revision would increase the usefulness of the index to this report.

### Royal Hospital for Sick Children, Glasgow.

A record amount of work in every department marked the year 1923; and the forecast of the Directors, when the new hospital was built, that they would be able in one year to treat 5,000 children in the wards, has been much more than realised, the number last year being as many as 5,853. Great, however, as is its present work, the hospital must be progressive; there can be no standing still. Accordingly, the Directors find it necessary to proceed with additions which will complete the original plans of the hospital as well as to make several further extensions, and an appeal for £60,000 for the purpose is now being issued. The complete financial transactions of the year resulted in an addition to capital of £5,186. The average daily cost of each in-patient was reduced from 7s. 6d. to 7s. 3d. We do not find stated in the Report the number of beds available; it appears to be between 250 and 300.

